# Twelve years of clinical practice guideline development, dissemination and evaluation in Canada (1994 to 2005)

**DOI:** 10.1186/1748-5908-4-49

**Published:** 2009-08-05

**Authors:** Jennifer Kryworuchko, Dawn Stacey, Nan Bai, Ian D Graham

**Affiliations:** 1 School of Nursing, Faculty of Health Sciences, University of Ottawa, 451 Smyth Rd, Room 3051, Ottawa, Ontario, K1H 8M5, Canada; 2 Canadian Medical Association, Ottawa, Ontario, Canada; 3 Knowledge Translation Portfolio, Canadian Institutes of Health Research, Ottawa, Ontario, Canada

## Abstract

**Background:**

Despite the growing availability of clinical practice guidelines since the early 1990's, little is known about how guideline development and dissemination may have changed over time in Canada. This study compares Canadian guideline development, dissemination, and evaluation in two six year periods from 1994–1999 and 2000–2005.

**Methods:**

Survey of guideline developers who submitted their clinical practice guidelines to the Canadian Medical Association Infobase (a Canadian guideline repository) between 1994 and 2005. Survey items included information about the developers, aspects of guideline development, and dissemination and evaluation activities.

**Results:**

Surveys were sent to the developers of 2341 guidelines in the CMA Infobase over the 12 year period, 1664 surveys were returned (response rate 71%). Of these, 730 unique guidelines were released from 1994–1999, and 630 were released from 2000–2005. Compared to the earlier period, more recent guidelines were being produced in English only. There has been little change in the type of organizations developing guidelines with most developed by provincial and national organizations. In the recent period, developers were more likely to report using computerized search strategies (94% versus 88%), publishing the search strategy (42% versus 34%), reaching consensus using open discussion (95% versus 78%), and evaluating effectiveness of the dissemination strategies (12% versus 6%) and the impact of the CPGs on health outcomes (24% versus 5%). Recent guidelines were less likely to be based on literature reviews (94% versus 99.6%) and were disseminated using fewer strategies (mean 4.78 versus 4.12).

**Conclusion:**

Given that guideline development processes have improved in some areas over the past 12 years yet not in others, ongoing monitoring of guideline quality is required. Guidelines produced more recently in Canada are less likely to be based on a review of the evidence and only about half discuss levels of evidence underlying recommendations. Guideline dissemination and implementation activities have actually decreased. Unfortunately, the potential positive impact on patient health outcomes will not be realized until the recommendations are adopted and acted upon.

## Background

In the 21^st ^century, clinical practice guidelines (CPGs) continue to be promoted as a means of improving the quality of patient care and patient health outcomes, reducing practice variation, and promoting more efficient use of health resources. Their potential benefits, however, are contingent on both rigorous guideline development processes that incorporate the best available evidence and successful implementation of guidelines into practice [1-4].

Canadian developers of 1446 guidelines released between 1994 and 1999 that were included in the Canadian Medical Association (CMA) InfoBase were surveyed to determine how guidelines were developed, disseminated, and evaluated; the analysis of these data were reported in an earlier publication[5]. At that time, the guideline development process was characterized by computerized searches of the literature, grading of the evidence in about half the guidelines, and consensus about recommendations using expert opinion and/or open discussion. Guideline developers largely disseminated their guideline via mailings direct to healthcare professionals or publications in professional newsletters or journals. Few evaluated their dissemination strategies or the impact of the guideline on health outcomes (6% and 5% respectively).

Reviews of systematic reviews about the effectiveness of various strategies to increase health care professionals use of research and practice guidelines published in the late 1990s indicated that dissemination of educational materials and didactic educational sessions had little or no effect on changing professional practice[6,7]. In the intervening years, considerable work has been done investigating the effectiveness of various dissemination and implementation strategies aimed at changing practitioners' practice. Grimshaw and colleagues' 2004 review of the effectiveness of guideline dissemination and implementation strategies supported the earlier findings that strategies such as reminders were potentially effective and resulted in moderate improvements in the process of care[8]. Educational outreach had modest effects although it was considered resource intensive and potentially costly[8]. Educational materials, audit and feedback, and patient directed interventions were less commonly evaluated but appeared to have "limited effect"[8]. A review of cluster randomized controlled trials revealed that passive strategies (e.g. mailings of printed educational material), contrary to conventional wisdom, may actually be useful for promoting the uptake of guidelines on their own by about 8%[3]. The same review showed that the median absolute improvement in performance across interventions was 14.1% for reminders; 7% for audit and feedback strategies; and 6% for strategies which included educational outreach[3].

Curious about whether there had been any changes in guideline development, dissemination, and evaluation activities in Canada over the past decade, the CMA continued to survey the developers of guidelines released during the period 2000–2005 and included in the CMA Infobase. This paper reports on how guidelines were developed, disseminated and evaluated in Canada between 1994 and 2005. We examined changes in these activities between the 6 year periods of 1994–1999 (earlier period) and 2000–2005 (recent period).

## Methods

### Design

The Canadian Medical Association surveyed Canadian developers of guidelines whose guidelines were included in the CMA Infobase between 2000 and 2005. Results were compared with the results from our previous survey of developers of guidelines released between 1994 and 1999[5]. The CMA Infobase, available to the public at , contains guidelines that are endorsed or developed by Canadian organizations within 5 years of the current date and are of interest to the CMA membership. At present, there are no quality criteria applied to screen the guidelines that are admitted to the repository. The CMA assigns English and French versions of bilingual guidelines a separate identifying number when each version is entered into the database and therefore both are included independently in the total number of guidelines in the database. The CMA automatically withdraws guidelines 5 years after their release. If a guideline is updated at any time, the original version is withdrawn and the update entered as a new guideline and the developers surveyed about the updated version. The database can be searched electronically by keyword, medical subject heading (MeSH), guideline developer, and recent additions to the database.

Developer organizations were classified as being one of seven types, in the same way as for the earlier analysis[5]. This classification was developed by the CMA. The seven types of organizations include (1) national professional organizations (e.g. Canadian Medical Association, Canadian Critical Care Trials Group), (2) provincial licensing bodies (e.g. College of Physicians and Surgeons of Manitoba), (3) provincial professional organizations (e.g. Alberta Medical Association), (4) government (e.g. Health Canada), (5) para-government (e.g. Cancer Care Ontario), and then consumer interest groups that were classified as (6) national health associations (e.g. Heart and Stroke Association of Canada) or (7) provincial health associations (e.g. Ontario Association of Medical Laboratories).

### Survey Instrument and Procedures

The survey was the same one as used in the previous study[5] (see additional file 1: Survey instrument). It was developed by the CMA in the early 1990s. No information on its reliability and validity is available, other than it has been used previously[5]. It elicited information about the process of guideline development including information about developers, the nature of the evidence-base, and how consensus was reached about the evidence and consideration of the benefits and harms. Another set of questions also focused on the use of strategies such as passive dissemination (e.g. educational resources mailed, published in journals or newsletters, or dissemination using computer technology like email or internet), educational strategies (e.g., CME activities, conferences or workshops), implementation activities (e.g., local opinion leaders, academic detailing, integration in licensing examinations, reminders, audit and feedback), and evaluation (e.g., impact of the guideline on health outcomes). Fortunately, no changes were made to the survey over the years which allows for direct comparison of the two time periods. Data on the number of guideline development committee members, the process use to select membership, and the committee member characteristics were not available for analysis for the 2000–2005 period. As part of the CMA's routine verification process, the survey was sent to all guideline developers when developers submitted a guideline to the CMA Infobase. The survey was returned by mail.

### Analysis

To examine changes in guideline development, dissemination, and evaluation over time, the year of release of the guidelines was arbitrarily dichotomized into two six year periods 1994–1999 (previously published by Graham and colleagues[5]) and 2000–2005. To investigate whether the volume of guidelines a developer produces is related to guideline development, dissemination or evaluation activities, we divided developers into two groups based on the median number of guidelines they had deposited to the CMA Infobase. Frequent guideline developers were those that had 4 or more guidelines in the Infobase and infrequent developers were those with 3 or fewer guidelines in the Infobase. Means and 95% confidence intervals were calculated for continuous variables. The results were analyzed descriptively using SPSS version 12 (SPSS Inc., Chicago, IL, USA).

## Results

### Developers

Of 2341 guidelines included in the CMA Infobase between 1994 and 2005, completed surveys were received for 1664 guidelines (71%). There were 1446 guidelines listed in the Infobase for the earlier period 1994–1999 and 895 guidelines listed for the recent period 2000–2005. As with the previous study[5], the response rate was based on the number of listed guidelines in the database. Since the CMA Infobase lists English and French versions of bilingual guidelines separately and assigns each version a unique identification number, we maintained this approach and therefore calculated the response rate in the same way that the Infobase catalogues the guidelines. Hence, the surveys from developers of bilingual guidelines, which gave information about two guidelines in the Infobase, were counted twice. Therefore, the response rate was 70% for the earlier period 1994–1999 (1012/1446) and 73% for the recent period 2000–2005 (652/895). However, for our analysis, we considered the data for each unique guideline only once (regardless of the number of translated versions) and therefore analyzed 730 unique guidelines released between 1994–1999[5] (earlier guidelines) and 630 unique guidelines released between 2000–2005 (recent guidelines). The decline in guidelines noted in 2004 and 2005 could represent a downward trend or variability in the way guidelines were produced and updates added to the CMA Infobase. We do not know whether this trend continued since 2005.

Over the 12 year period, the 1360 unique guidelines were developed by a total of 96 different guideline developers: 75 different guideline developers for the 1994 to 1999 period and 56 for the 2000 to 2005 period. Over 80% of the guidelines in both periods were produced by national professional, para-government, government, or licensing bodies (Table 1). Over both time periods there were1032 guidelines released in English only, 24 released in French only, and 304 with English and French versions. Recent guidelines were published in English only and fewer guidelines had a French version. There were no uniquely French guidelines in the recent time period.

**Table 1:** Characteristics of guidelines and developers

	Total guidelines (n = 1360)		1994–1999 guidelines (n = 730)		2000–2005 guidelines (n = 630)	
	
	N	%	n	%	n	%
*Type of organization*						

National professional (e.g. Canadian Medical Association)	450	33.1	254	34.8	196	31.1

Para-government (e.g. Cancer Care Ontario)	313	23.0	177	24.2	136	21.6

Licensing (e.g. College of Physicians and Surgeons of Manitoba)	180	13.2	116	15.9	64	10.2

Government (e.g. Canadian Task Force on the Periodic Health Exam)	243	17.9	113	15.5	130	20.6

Provincial medical (e.g. Alberta Medical Association)	62	4.6	29	4.0	33	5.2

National health association (e.g. Heart and Stroke Foundation of Canada)	88	6.5	24	3.3	64	10.2

Provincial health association (e.g. Ontario Association of Medical Laboratories	24	1.8	17	2.3	7	1.1

*Language*						

English only	1032	75.9	424	58.1	608	96.5

French only	24	1.8	24	3.3	0	0

Bilingual	304	22.4	282	38.6	22	3.5

*Date of publication*						

1994	183	13.5				

1995	109	8.0				

1996	122	9.0				

1997	114	8.4				

1998	129	9.5				

1999	73	5.4				

2000	113	8.3				

2001	130	9.6				

2002	124	9.1				

2003	123	9.0				

2004	90	6.6				

2005	50	3.7				

Of 1360, the total number of guidelines produced ranged from 1 to 167 per guideline developer (mean = 14.17, median = 3, mode = 1, SD = 30.08). Frequent developers, those who developed 4 or more guidelines, comprised 41 of the 96 guideline developers (42%) and were responsible for developing 1275 (93.8%) guidelines (Table 2). Infrequent developers, those who developed 3 or less guidelines, were 55 of the 96 developers but were only responsible for 85 (6.3%) of the 1360 guidelines. In the twelve year time period, 16 developer organizations were responsible for producing 78% of the guidelines (1068/1360). From 1994 – 1999, the 730 guidelines were produced by 75 different organizations, who produced between 1 and 96 guidelines (mean = 9.73, median = 3, SD = 20.05). In this earlier time period, 16 developers were responsible for 78% of guidelines (575/730). In the more recent time period, 630 guidelines were produced by 56 different organizations who produced between 1 and 79 guidelines (mean = 11.25, median = 2, SD = 19.534). As well, in this recent time period, 11 developers were responsible for 78% of the guidelines (490/630).

**Table 2:** Differences between infrequent and frequent developers over twelve years

	Total developers n = 96		Difference
	
	Infrequent developers n = 55 % (95% CI)	Frequent developers n = 41 % (95% CI)	%
Scientific literature reviewed	94.1 (89.0–99.2)	97.3 (96.4–98.2)	3.2

Computerized literature search	76.6 (66.9–86.3)	91.6 (90.1–93.2)	15.0

Search strategy stated in guideline	37.1 (25.5–48.8)	37.3 (34.6–40.1)	0.2

Quality of evidence graded	38.1 (27.5–48.7)	51.6 (48.8–54.4)	13.5

Passive dissemination strategies	90.6 (84.3–96.9)	91.4 (89.8–92.9)	0.8

Educational strategies	45.8 (35.1–56.7)	48.3 (45.5–51.1)	2.5

Active implementation strategies	29.4 (19.5–39.3)	33.0 (30.4–35.6)	3.6

Effectiveness of dissemination/implementation strategies formally evaluated	3.7 (0–7.8)	9.2 (7.6–10.8)	5.5

Plan to evaluate dissemination/implementation in future	29 (17.4–40.7)	44.4 (41.2–47.6)	15.4

Formally evaluated impact on health outcomes	6.1 (0.8–11.4)	14.1 (12.1–16)	8.0

Plan to evaluate impact on health outcomes in future	32.3 (20.3–44.2)	43.9 (40.7–47.1)	11.6

Companion consumer version	12.9 (5.7–20.2)	25.4 (23–27.8)	12.5

Plan to produce consumer version in future	7.1 (1.5–12.6)	20.5 (18.3–22.7)	13.4

### Guideline development characteristics

There have been statistically significant changes in characteristics of the guideline development process between the two periods (Table 3). In the recent period, more guideline developers reported using a computerized search (93.6% up from 87.9%), stated the search strategy in the document (41.7% up from 33.5%), and used open discussion to reach consensus about the recommendations (95.2% up from 78.4). Fewer guidelines were based on a review of the literature (94.3% down from 99.6%), and fewer used structured processes (such as Delphi technique) to reach consensus (1.3% down from 12.5%). While the proportion of guidelines that explicitly graded the quality of the evidence supporting the recommendations declined (46.9% down from 53.9%), this difference was not statistically significant.

**Table 3:** Change in guideline development process

	Year of development n = 1630 guidelines		Change
	
	1994–1999 n = 730 % of guidelines (95% CI)	2000–2005 n = 630 % of guidelines (95% CI)	%
Scientific literature reviewed	99.6 (99.1–100)	94.3 (92.5–96.1)	-5.3

Computerized literature search	87.9 (85.6–90.4)	93.6 (91.7–95.5)	+5.7

Search strategy stated in guideline	33.5 (29.9–37.0)	41.7 (37.8–45.7)	+8.2

Quality of evidence graded	53.9 (50.2–57.5)	46.9 (42.9–51.0)	-7.0

*Consensus reached by:*			

Open discussion	78.4 (75.4–81.4)	95.2 (93.5–96.9)	+16.8

Structured process (e.g. Delphi or nominal technique)	12.5 (10.0–14.9)	1.3 (0.4–2.2)	-11.2

Other (e.g. Expert opinion)	9.1 (7.0–11.2)	3.5 (2.1–5.0)	-5.6

When the development process of frequent and infrequent developers in the CMA Infobase were compared, more frequent producers were more likely have conducted a computerized literature search (91.6% compared to 76.6%) and graded the quality of evidence used in the guideline (51.6% compared to 38.1%)(Table 2).

### Knowledge translation strategies used to promote the uptake of guidelines

More recently, guideline developers engaged in significantly fewer dissemination and implementation activities per guideline than in the earlier period (Table 4). On average there was a small but statistically significant decrease in the total number of knowledge translation dissemination strategies used per guideline (mean = 4.12 down from 4.78). The mean number of passive, educational, and active strategies used per guideline was less (only the decline in the number of passive strategies was statistically significant).

**Table 4:** Knowledge translation strategies employed per guideline

*Strategies used per guideline*	Year of development n = 1360 guidelines		Change
	
	1994–1999 n = 730 mean strategies (95% CI)	2000–2005 n = 630 mean strategies (95% CI)	mean
Total knowledge translation strategies	4.78 (4.58–4.98)	4.12 (3.89–4.36)	- 0.66

Passive strategies	3.38 (3.27–3.50)	2.94 (2.79–3.09)	- 0.44

Educational strategies	0.74 (0.68–0.80)	0.64 (0.58–0.71)	- 0.10

Implementation strategies	0.65 (0.57–0.72)	0.53 (0.45–0.61)	- 0.12

An examination of the actual strategies used per guideline (Table 5) reveals that overall, the proportion of guidelines using at least one passive strategy such as mailings, publishing newsletters or journals, and using computer technology to disseminate the guidelines, decreased by 15% between the two time periods. The decrease in passive strategies was not offset by an increase in either educational or active implementation strategies (e.g using opinion leaders, academic detailing, reminder systems, etc). Fewer education (58.4% down from 64.7%) and active implementation (29.5% down from 35.6%) strategies were used in the recent period although the declines were not statistically significant. More developers reported developing a companion document in a format designed for consumers in the recent period (28.4% up from 21.3%).

**Table 5:** Passive dissemination, education, and active implementation strategies

*Strategy*	Year of development n = 1360 guidelines		Change
	
	1994–1999 n = 730 % of guidelines (95% CI)	2000–2005 n = 630 % of guidelines (95% CI)	%
*Passive dissemination strategies (at least 1)*	98.1 (97.1–99.1)	83.5 (80.6–86.4)	-14.6

Direct mailing to membership/conference participants	80.3 (77.4–83.2)	70.5 (66.9–74.1)	-9.8

Publishing in newsletters or journals	75.8 (72.6–78.9)	63.5 (59.7–67.3)	-12.3

Direct mailing to others	73.3 (70.1–76.5)	63.5 (59.7–67.3)	-9.8

Computer technology	62.3 (58.8–65.9)	54.6 (50.7–58.5)	-7.7

*Educational strategies (at least 1)*	64.7 (61.2–68.1)	58.4 (54.6–62.3)	-6.3

Providing guideline information to patients or consumers	47.3 (43.6–50.9)	42.4 (38.5–46.3)	-4.9

Educational or continuing medical education (CME) activities	50.2 (46.6–53.8)	43.7 (39.8–47.5)	-6.5

Organization/sponsorship of conferences or workshops	24.1 (21.0–27.2)	21.1 (17.9–24.3)	-3

*Active implementation strategies (at least 1)*	35.6 (32.1–39.1)	29.5 (25.9–33.1)	-6.1

Training and support of people who have educational or administrative influence (local opinion leaders)	16.7 (14.0–19.4)	14.6 (11.8–17.4)	-2.1

Face to face visits at practitioners' offices (academic detailing/outreach)	15.6 (13.0–18.3)	12.7 (10.1–15.3)	-2.9

Guideline reminder systems (manual or computer)	15.2 (12.6–17.8)	11.9 (9.4–14.4)	-3.3

Training or support for audit and feedback	13.0 (10.6–15.5)	12.2 (7.8–12.5)	-0.8

Integration of guideline into recertification or licensing examinations	2.5 (1.3–3.6)	1.9 (0.8–2.9)	-0.6

Administrative strategies such as the design of laboratory or x-ray forms	2.3 (1.2–3.4)	2.2 (1.0–3.4)	-0.1

Other (e.g. media campaign)	4.8 (3.3–6.4)	4.1 (2.6–5.7)	-0.7

Examining KT strategies by frequent and infrequent developers, we note that guidelines produced by frequent developers employed active, passive and educational strategies to promote their guideline at similar rates to the infrequent developers (Table 2). Frequent developers were twice as likely to both formally evaluate the guideline to determine its impact on health outcomes (14.1% compared to 6.1%) and design a companion document specifically designed for consumers (25.4% compared to 12.9%). As well, frequent developers indicated more often that they intended to produce such a consumer document in future (20.5% compared to 7.1%).

### Guideline evaluation activities

There was an increase in guideline developers who reported either evaluating the effectiveness of their dissemination strategies or the impact of the guideline on health outcomes in the recent period (Table 6). Developers reported that the effectiveness of dissemination or implementation strategies had been evaluated more often for the recent guidelines (12.2% up from 6.1%). Similarly, the proportion of guidelines formally evaluated to determine their impact on health outcomes also increased (23.8% up from 5.1%).

**Table 6:** Guideline evaluation activities

*Evaluation Activities*	Year of development n = 1360 guidelines		Change
	
	1994–1999 n = 730 % of guidelines (95% CI)	2000–2005 n = 630 % of guidelines (95% CI)	%
Effectiveness of dissemination/implementation was formally evaluated	6.1 (4.4–7.8)	12.2 (9.53–14.8)	+6.1

Plan to evaluate dissemination/implementation in future	59.8 (55.7–63.9)	22.7 (18.8–26.7)	-37.1

Guideline impact on health outcomes was formally evaluated	5.1 (3.5–6.7)	23.8 (20.4–27.3)	+18.7

Plan to evaluate guideline impact on health outcomes in future	51.4 (47.3–55.6)	32.6 (28.3–37.1)	-18.8

Companion document available for consumers	21.3 (18.4–24.4)	28.4 (24.9–31.9)	+7.1

Intend to produce consumer version in future	24.3 (21.1–27.4)	14.3 (11.6–17.0)	-10

In order to more fully appreciate the decline in the use of dissemination and implementation strategies, we looked at their use by different guideline developers. There was a decline in the use of passive strategies by national professional organizations (71.4% down from 98.4%) and national health organizations (50% down from 95.8%). While licensing bodies were using less educational (34.4% down from 84.5%) and less active implementation strategies (18.8% down from 80.2%), government organizations were using more of both educational (56.9% up from 12.4%) and active implementation strategies (45.4% up from 2.7%). Provincial organizations also increased their use of educational strategies (14.3% up from 0%).

Although guidelines produced by developers with 4 or more guidelines in the CMA Infobase were not more likely to evaluate the effectiveness of the dissemination and implementation strategies, they were more likely to plan to do so in future (44.4% compared to 29%)(Table 2). More often, frequent developers formally evaluated guideline impact on health outcomes (14.1% compared to 6.1%).

## Discussion

Guideline development in Canada, as elsewhere, is undertaken by many different organizations and so it is challenging to know "who is doing what." Surveying guideline developers about their processes of guideline development and implementation and doing so over time, offers a unique glimpse in the guideline industry in Canada and how it is evolving. We are unaware of any other national longitudinal data revealing trends over time in the practices of major guideline developers. Our findings are also unique in that we report on guideline developers' efforts to increase the use of guidelines and evaluate their impact. These are areas for which there are very few data in the literature despite the critical role of implementation strategies in facilitating the uptake of guidelines. Furthermore, without the adoption of guidelines by health providers there will certainly be no impact on health status or health system outcomes, the ultimate purpose of developing guidelines in the first place.

Comparing guidelines released and included in the CMA Infobase from 1994–1999 and 2000–2005, revealed that 100 fewer guidelines were deposited in the CMA Infobase in the recent 6 year period and 19 fewer guideline developers submitted their guidelines to the Infobase. While this may suggest that guideline development in Canada may be slowing, there is no way to know whether this reflects the development of fewer guidelines or whether guideline developers were depositing their guidelines in the CMA Infobase less often. For both time periods, national professional, para-government and government associations and agencies were the dominant producers of guidelines.

Over the 12 year period, guideline developers in Canada increasingly submitted guidelines in English only. More recent guidelines were 6% more likely to have conducted a computerized search of the literature, 8% more likely to have stated the search strategy in the guideline document, and 17% more likely to have reached consensus via open discussion than guidelines in the earlier period (11% reduction in the use of structured processes to reach consensus). The greater reliance on computer searching for the evidence and greater transparency about the search strategy in the more recent period is positive but it is not known whether this translated in higher quality guidelines.

Of concern is the fact that less than half the guidelines in the recent period graded the quality of the evidence and 6% did not even review the scientific literature and both at lower rates than that of guidelines produced in the earlier period. While we did not assess the quality of the guidelines in the CMA Infobase in this study, previous work has revealed that the quality of drug guidelines in this database was less than optimal[9] and given the limited changes in guideline development reported between the two periods, there is little reason to expect that the quality of Canadian Guidelines has vastly improved over the 12 year study period.

Given the international efforts such as the AGREE Collaboration[1] to improve the quality of reporting of practice guidelines and the GRADE working group[10] to encourage consensus on approaches to grading of the evidence, the timing may be right to encourage and support Canadian guideline developers to improve the rigor of the methods used to develop their guidelines and their reporting.

In terms of guideline developer knowledge translation activities over the two time periods, there has been a small but significant decrease in the total number of dissemination and implementation strategies employed per guideline. This was largely due to the use of fewer passive dissemination strategies in the more recent period. One hypothesis for the decline might be growing awareness of early evidence that passive dissemination was ineffective at changing professional behaviour[6,7]. More recent evidence[3] is suggesting that there may actually be value in passive dissemination since it is inexpensive and may be as effective as more costly and labour intensive approaches such as audit and feedback[3,8]. While the evidence continues to indicate that interactive education approaches and more active implementation strategies can be effective in changing professional behaviour[8,11-13], there has also been small non significant declines in the use of these activities in the more recent period which may suggest developers are unaware of, or choosing to ignore, this evidence.

If the benefits of the guidelines produced are to be achieved, guideline developers and their stakeholders should reconsider their dissemination and implementation activities and how to work together to better encourage the adoption of their guidelines into routine practice. Based on the findings of Grimshaw and colleagues, it is reasonable to continue using passive dissemination but also important to use more targeted implementation strategies aimed at overcoming contextual barriers to implementation and embedding guidelines within organizational structures such as documentation and ordering systems[8]. As these activities have resource implications, it will be important for guideline developers and KT researchers to consider the cost-effectiveness of dissemination and implementation strategies in the future.

It is encouraging that in the more recent period, the effectiveness of dissemination and implementation activities is being evaluated in twice as many guidelines (12.2% vs. 6.1%). Since there remains considerable room for greater research on KT strategies, it is unfortunate that fewer developers reported intending to undertake such evaluations in the future. The proportion of guidelines whose developers report formally evaluating the impact of their guideline on health incomes has increased substantially over the 12 year period from 5% to 24% of guidelines. Data on the positive health outcomes of guidelines may be useful for encouraging others to incorporate guidelines into their healthcare decision making. The opportunity to evaluate the impact of a guideline on health outcomes may also provide a safe forum for potential adopters to try the guideline, to contextualize the recommendations of the guideline for their clinical setting and to support implementation under temporary research conditions. Evaluation research can be considered an active implementation strategy, especially where the changes in clinical practice recommended in the guideline are sustained.

Our comparison of developers who submitted three or fewer, or 4 or more, guidelines to the CMA Infobase over the 12 year period revealed some interesting findings that will need to be confirmed by future research. Guidelines produced by more experienced guideline developers were more likely to have done a computerized search of the literature, graded the quality of the evidence, planned to formally evaluate the dissemination/implementation strategies they use, formally evaluated the impact of the guideline on health outcomes, and had a companion document for consumers. There were no differences in terms of the dissemination and implementation activities undertaken by the two groups. One interpretation of these findings is that the volume of guidelines produced by a developer may be important and related to higher guideline quality and evaluation but not KT activities. Graham and colleagues previously found lower quality in guidelines developed by government, para-government or professional organizations compared to those developed by other types of guideline developers[9]. Consequently, more research is needed to understand the relationship between guideline quality and characteristics of guideline developers.

### Limitations

These findings should be considered within the limitations of the study. First, the survey data were self-reported by the guideline developers and were not objectively verified. However, the information provided in the survey (information about the guideline development process) is available on the CMA Infobase with the guideline and therefore makes verification of survey responses possible. Another consideration was that the survey was also sent only after the guideline had been accepted for inclusion in the CMA Infobase rather than as part of the process of accepting the guideline. Both of these factors may have encouraged guideline developers to accurately report their responses. Furthermore, since more quality indicators remain unmet in the recent time period, the change in response between the two study periods is likely an accurate reflection of their activities.

Another limitation relates to the questions used to assess the quality of guideline development. In the years since the survey was developed, the AGREE Collaboration[1] has developed criteria for assessing guideline quality. Although the CMA Infobase survey items address many of the same concepts as the AGREE Instrument, the time may be right for the CMA to adopt or adapt the AGREE instrument to survey guideline developers about the quality of their development processes.

A third limitation is that conclusions can only be drawn about the guidelines and their developers that were deposited into the CMA Infobase during the study period. We have no way of knowing what proportion of Canadian guidelines is deposited in the Infobase. It is possible that guideline developers producing guidelines in French may not be submitting them to the CMA Infobase since only about one-third of Quebec physicians are members of the CMA. There are also other repositories of guidelines for non-physician health care providers in Canada (for example, RNAO's Best Practice Guidelines at ). However, CMA believes that the database represents the majority of CPGs published in English Canada since they have built a comprehensive searching and screening strategy that adds to what developers' submit: they search various databases (notably Medline) and websites regularly and hand search major medical journals for guidelines of interest to physicians. The CMA also believes that the guidelines that are not identified in this process are most likely published by some very small specialty groups. Finally, it is likely that developers will deposit their guidelines here since this is the primary repository for guidelines targeting Canadian physicians, who can access the CMA Infobase at no cost.

## Conclusion

Guideline development processes in Canada have improved in some areas over the past 12 years yet not in others. Guideline dissemination and implementation activities have actually decreased. Therefore, guideline developers might benefit from having resources on best practices for guideline development. CMA have started this process with the updating of Handbook on Practice Guidelines [14]. Encouraging guideline developers who have developed processes that ensure high quality guidelines to share these practices with other smaller or less resourced developers may be another means of improving the quality of Canadian guidelines. Ultimately the putative benefits of guideline recommendations will not be reaped until the recommendations are adopted and acted upon. Knowledge translation researchers and guideline developers must do more work to determine the most effective strategies for promoting the use of specific guidelines with specific health care providers in particular settings.

## Competing interests

The authors declare that they have no competing interests. Nan Bai is an information specialist at CMA in charge of Infobase.

## Authors' contributions

JK participated in the design of the study, completed the statistical analysis, and drafted the manuscript. IDG conceived of the study, provided input into the design of the survey, guided the analysis and helped draft the manuscript. DS contributed to the interpretation of the results and drafting of the manuscript. NB provided the data and contributed to interpreting the results. All authors read and approved the final manuscript.

## Supplementary Material

Additional file 1 **Survey instrument**. Survey developed by the CMA in the early 1990s and used to report on guidelines in the CMA Infobase from 1994–2005. Click here for file
