# Supplementary material for: Twelve years of clinical practice guideline development, dissemination and evaluation in Canada (1994 to 2005)
Source: Implement Sci. 2009 Aug 5;4:49. doi: 10.1186/1748-5908-4-49 (PMC2731072; doi:10.1186/1748-5908-4-49)
Supplement: Additional file 1 — Survey instrument. Survey developed by the CMA in the early 1990s and used to report on guidelines in the CMA Infobase from 1994–2005. [file 1748-5908-4-49-S1.pdf]

Survey instrument

1. What is the name of the committee or working group who was responsible for the actual development of the guideline?
2. How many members sit on this committee or working group?
3. Was there a formal process by which members were selected? ☐Yes ☐ No
4. Which of the following categories of individuals sit on this committee?
   Please check all that apply?
   - ☐ Physicians ☐ Specialists ☐ General practitioners
   - ☐ Representatives of specialty societies or organized medicine, e.g. CMA, OMA
   - ☐ Other health care professionals
   - ☐ Patients/consumers
   - ☐ Government representatives
   - ☐ Others (please specify): ____
5. Was scientific literature reviewed during the development of this guideline? ☐Yes ☐ No
6. Was a computerized search of the literature undertaken? ☐Yes ☐ No
7. Is the search strategy stated in the guideline? ☐Yes ☐ No
8. Was any attempt made to grade the quality of evidence using a grading system such as the ones suggested by the Canadian Task Force on the Periodic Health Examination or the criteria proposed by Sackett et al (1989)? ☐Yes ☐ No
9. How was consensus reached about values or judgments during the development of this guideline? (Please check one option only.)
   - ☐ On the basis of expert opinion and open discussion
   - ☐ On the basis of a structured process such as Rand approach (the Delphi technique)
   - ☐ Other (please specify):____
10. Were the benefits, harms, and costs of the potential intervention, and an explicit estimate of the probability of each outcome, considered? ☐Yes ☐ No
11. Have you undertaken any of the following dissemination or implementation activities with this guideline? (Please check all that apply.)
    - ☐ Direct mailing of guideline to membership or conference participants
    - ☐ Direct mailing of guideline to others
    - ☐ Dissemination of guideline via computer technology

- ☐ Publishing in newsletters or journals
- ☐ Organization / sponsorship of conferences or workshops
- ☐ Educational or CME activities
- ☐ Integration of guideline into recertification or licensing examinations
- ☐ Face to face visits at practitioner's office(Academic detailing or outreach visits)
- ☐ Training and support of people who have educational or administrative influence (local opinion leaders)
- ☐ Training and support for audit and feedback
- ☐ Guideline reminder systems (manual or computerized)
- ☐ Administrative strategies such as the design of lab or X-ray forms
- ☐ Providing information about the guideline to patients /consumers
- ☐ Other (Please specify): ____

12. Has the effectiveness of the dissemination or implementation strategies used been formally evaluated?
    - ☐ Yes. Please send us a copy.
    - ☐ No. Are there evaluation plans for the future? ☐Yes ☐ No If yes, when?_________
13. Has the guideline been formally evaluated to determine its impact on health?
    - ☐ Yes. Please send us a copy.
    - ☐ No. Are there evaluation plans for the future? ☐Yes ☐ No If yes, when?_________
14. Has your or any other organization produced a companion document about this guideline in a format specifically designed for patients/consumers?
    - ☐ Yes. Please send us a copy.
    - ☐ No. Are you intending to produce one? ☐Yes ☐ No If yes, when?_________

Thank you for completing this questionnaire.
